# Preparation, Characterization, and Adsorption Performance of an Interlayer-Expanded PPy/Maghnite–Cu^2+^ Nanocomposite for Methylene Blue Removal

**DOI:** 10.3390/polym18091052

**Published:** 2026-04-26

**Authors:** Mohamed Amine Bekhti, Faiza Zahaf, Ouiddad Saiah, Abdelghani Baltach, Dursun Murat Sekban, Ecren Uzun Yaylacı, Murat Yaylacı

**Affiliations:** 1 Laboratory of Materials, Applications and Environment, Faculty of Exacte Sciences, Mustapha Stambouli University of Mascara, BP 763, Mascara 29000, Algeria; mohamed.bekhti@univ-mascara.dz (M.A.B.); faiza.zahaf@univ-mascara.dz (F.Z.); 2 Laboratory of Organic, Macromolecular, and Materials Chemistry, Faculty of Exacte Sciences, Mustapha Stambouli University of Mascara, BP 763, Mascara 29000, Algeria; widad.saiah@univ-mascara.dz; 3 Department of Mechanical Engineering, University of Ibn Khaldoun, BP 78, Tiaret 14000, Algeria; abdelghani.baltach@univ-tiaret.dz; 4 Mechanics of Materials, Energy and Environment Laboratory (L2M2E), Mascara 29000, Algeria; 5 Department of Marine Engineering Operations, Karadeniz Technical University, 61080 Trabzon, Türkiye; msekban@ktu.edu.tr; 6 Trabzon Teknokent, WMS Engineering Services Industry Trade Limited Company, 61080 Trabzon, Türkiye; 7 Faculty of Fisheries, Recep Tayyip Erdogan University, 53100 Rize, Türkiye; ecren.uzunyaylaci@erdogan.edu.tr; 8 Department of Civil Engineering, Recep Tayyip Erdogan University, 53100 Rize, Türkiye; 9 Turgut Kıran Maritime Faculty, Recep Tayyip Erdogan University, 53900 Rize, Türkiye; 10 Dijitalpark Teknokent, Murat Yaylacı-Luzeri R&D Engineering Company, 53100 Rize, Türkiye

**Keywords:** PPy/maghnite–Cu^2+^ nanocomposite, maghnite clay, polypyrrole, intercalated nanocomposite, methylene blue adsorption, hybrid adsorbent, wastewater treatment

## Abstract

The development of efficient and low-cost adsorbents for dye-contaminated wastewater remains an important challenge in environmental remediation. In this study, an interlayer-expanded polypyrrole/maghnite–Cu^2+^ nanocomposite (PPy/Mag–Cu^2+^) was successfully synthesized through purification of raw maghnite, sodium activation, Cu^2+^ ion exchange, and in situ oxidative polymerization of pyrrole. The obtained hybrid was characterized by X-ray fluorescence, X-ray diffraction, Fourier-transform infrared spectroscopy, UV–Vis spectroscopy, scanning electron microscopy, and cyclic voltammetry. The results confirmed the successful incorporation of Cu^2+^ and polypyrrole while preserving the layered aluminosilicate framework. XRD analysis revealed a progressive increase in basal spacing from 17.49 Å for raw maghnite to 25.95 Å for the final nanocomposite, indicating effective intercalation and formation of an expanded hybrid structure. The adsorption performance of PPy/Mag–Cu^2+^ was evaluated for methylene blue removal under batch conditions. Adsorption was strongly influenced by contact time, pH, and initial dye concentration, with equilibrium reached after approximately 80 min and optimum removal at pH 9. Equilibrium data were best fitted by the Langmuir model, with a maximum monolayer adsorption capacity of 43.66 mg g^−1^, while kinetic data followed the pseudo-second-order model. These findings demonstrate that PPy/Mag–Cu^2+^ is a promising and cost-effective hybrid adsorbent for cationic dye removal from aqueous media.

## 1. Introduction

The persistent flow of synthetic dyes into water systems is a severe environmental hazard related to rising industry. Among these pollutants, methylene blue (MB) is extensively exploited in the textile, pharmaceutical, and printing industries thanks to its high stability and large coloring power. However, its persistence, limited biodegradability, and probable toxicity demand efficient removal prior to wastewater disposal [1,2,3,4]. Even at low concentrations, MB reduces light penetration in aquatic bodies, decreases photosynthetic activity, and generates ecological imbalance [4,5]. Among the different water treatment methods, adsorption remains one of the most successful and economically practical ways owing to its simplicity, high effectiveness, and adaptability [6,7,8]. Recent research addresses the fabrication of nanostructured adsorbents with enhanced surface area and tunable surface chemistry to improve dye removal performance [9,10,11]. In addition, current material design strategies increasingly consider structural accessibility, interfacial functionality, and broader application potential when developing high-performance adsorbent systems [12,13,14,15,16,17,18]. In this context, layered clay minerals such as montmorillonite have received great attention because of their high cation-exchange capacity, swelling ability, layered structure, and natural abundance [19,20,21]. Nevertheless, pure clays often display insufficient adsorption capacity toward bulky organic dyes due to restricted interlayer accessibility and inadequate active sites [22,23]. Surface engineering approaches, including ion exchange with transition metals and interlayer modification, have been reported to considerably increase adsorption efficacy by increasing basal spacing and introducing new coordination sites [24,25,26]. However, conventional clay modification techniques often remain limited to cation substitution or surface alteration alone, which may not sufficiently improve the accessibility of internal adsorption sites for relatively large dye molecules. In this respect, interlayer expansion is scientifically important because it enlarges the gallery space, facilitates mass transfer toward the internal active sites, and creates a more accessible host structure for the incorporation of functional species while preserving the layered clay framework. Copper-modified clays, in particular, display increased affinity for nitrogen-containing dyes via electrostatic attraction and coordination interactions [27]. Among the transition metal ions commonly used for clay modification, Cu^2+^ is particularly attractive because it can provide effective coordination with nitrogen-containing molecules while also supporting interlayer modification without altering the layered framework. This makes Cu^2+^ a suitable choice in the present system compared with more conventional transition metal exchange strategies that do not simultaneously emphasize both coordination activity and interlayer accessibility. In parallel, conductive polymers such as polypyrrole (PPy) have emerged as possible materials for environmental remediation. Their π-conjugated backbone, redox activity, and strong interaction capabilities with aromatic molecules enable diverse adsorption mechanisms, including electrostatic attraction, π–π stacking, and redox-assisted processes [28,29]. However, pure conducting polymers may suffer from aggregation and limited mechanical stability. The mixing of layered clays with conductive polymers offers an effective way to make multifunctional organic–inorganic nanocomposites with synergistic features [30]. While past studies have addressed either polymer-coated clays or metal-exchanged clays, few have reported a deliberately interlayer-engineered tri-component system that combines Cu^2+^-exchanged montmorillonite with in situ intercalated electroactive polypyrrole within the same layered architecture [31]. In this work, Algerian maghnite (montmorillonite) was purified, sodium-activated, and further altered by controlled Cu^2+^ exchange, followed by in situ oxidative polymerization of pyrrole within the expanded interlayer galleries. Therefore, the novelty of the present PPy/Maghnite–Cu^2+^ composite lies in the stepwise construction of an interlayer-expanded hybrid structure that differs from previously reported polymer-clay composites by integrating metal exchange, gallery expansion, and polymer intercalation in a single material design. This stepwise method seeks to construct a structurally integrated PPy/Maghnite–Cu^2+^ nanocomposite, integrating an increased interlayer gap, negatively charged silicate layers, copper coordination sites, and a π-conjugated redox-active polymer network. Such a multifunctional design is expected to promote cooperative electrostatic attraction, π–π stacking, coordination bonding, and redox-assisted interactions, therefore improving methylene blue adsorption efficiency.

## 2. Experimental Procedure

### 2.1. Materials

Pyrrole monomer (Py, 99%) was purchased from Sigma-Aldrich (St. Louis, MO, USA) and purified prior to use when necessary. Ammonium persulfate ((NH_4_)_2_S_2_O_8_, APS), used as an oxidizing agent for the in situ polymerization of pyrrole, was supplied by Merck (Darmstadt, Germany) and used without further purification. Dimethyl sulfoxide (DMSO) and perchloric acid (HClO_4_, Suprapur grade) were obtained from Sigma-Aldrich (St. Louis, MO, USA) and Merck (Darmstadt, Germany), respectively. Copper sulfate (CuSO_4_) and sodium chloride (NaCl) were employed for the preparation of Cu^2+^- and Na^+^-exchanged clay derivatives. The clay mineral used in this study was montmorillonite, commercially known as Maghnite, collected from the Tlemcen region in western Algeria. Methylene blue (MB), used as the model cationic dye in the adsorption experiments, was selected to evaluate the removal performance of the synthesized nanocomposite in aqueous solution. All aqueous solutions were prepared using ultrapure water (18.2 MΩ·cm) produced by an ELGA LabWater Purelab Ultra system (ELGA LabWater, High Wycombe, UK). These materials were used for the purification and modification of Maghnite, followed by the synthesis of the interlayer-expanded PPy/Mag–Cu^2+^ nanocomposite and its application as an adsorbent for methylene blue removal from aqueous media. 

### 2.2. Preparation and Modification of Maghnite

#### 2.2.1. Purification and Activation with Sodium (Maghnite–Na^+^)

There are many impurities in raw maghnite, such as carbonates, quartz, cristobalite, iron oxides/hydroxides, and organic residues. These impurities can make adsorption and ion exchange less effective. A multi-step purification process was adopted to ensure reproducibility and preserve the structure. The purification and sodium activation procedure was carried out under the same operating conditions in independent preparations, and comparable clay fractions were consistently obtained. First, the clay was washed with distilled water to get rid of soluble salts and impurities that were only weakly bound. After that, a 10 g sample was ground in a ceramic ball mill for 20 min to reduce particle size and improve dispersion.

Subsequent dispersion–sedimentation cycles in distilled water enabled the isolation of the clay-rich fraction from denser mineral phases. After settling, the cleaned clay was dried at 150 °C for 24 h and stored in sealed glass containers. The purified clay was subjected to sodium activation to enhance its swelling and cation-exchange capabilities. This was accomplished by applying a concentrated NaCl solution to the material, which allowed Na^+^ ions to replace native interlayer cations. Under these conditions, a stable clay-rich sodium-activated fraction was consistently recovered after purification and sedimentation, indicating the reproducibility of the pretreatment procedure. The resulting Na-exchanged montmorillonite is hereafter denoted as Maghnite–Na^+^ (Mag–Na^+^).

#### 2.2.2. Copper Exchange (Maghnite–Cu^2+^)

Copper (II)-exchanged clay was prepared by dispersing Mag–Na+ in a 1 M CuSO_4_ solution under continuous magnetic stirring for 24 h at ambient temperature. After exchange, the material was collected by centrifugation and washed again with deionized water until the washings tested negative for chloride (using AgNO_3_) and sulfate (using Ba(NO_3_)_2_), confirming complete removal of residual ions. Maghnite–Cu^2+^ (Mag–Cu^2+^), the finished product, was dried at 110 °C for the entire night. Its elemental composition was then investigated by X-ray fluorescence (XRF) spectroscopy, and the results are provided in Table 1.

### 2.3. Synthesis of PPy/Maghnite–Cu^2+^ Nanocomposite Adsorbent

**Figure 1:**
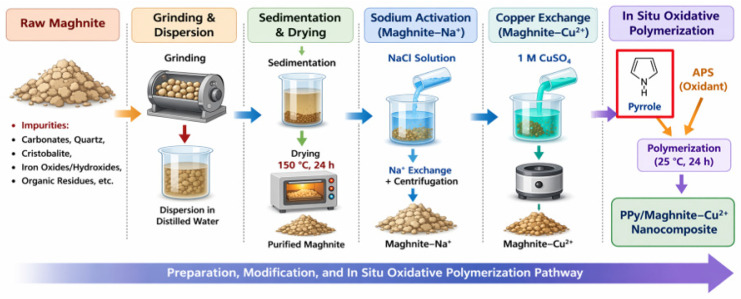
Preparation and modification of Maghnite clay and in situ oxidative polymerization for the synthesis of PPy/Maghnite–Cu^2+^ nanocomposite.

The polypyrrole/clay nanocomposite was formed via in situ oxidative polymerization of pyrrole in the presence of the copper-modified clay, as depicted schematically in Figure 1, with pyrrole represented as a five-membered heterocyclic monomer.

In a typical method, 0.5 g of Mag–Cu^2+^ was dispersed in a 0.22 M pyrrole solution and agitated magnetically for 2 h at room temperature to promote monomer intercalation into the clay interlayer gaps. Polymerization was then initiated by the dropwise addition of 100 mL of a pre-cooled 0.22 M APS solution, corresponding to an APS to pyrrole molar ratio of 1:1, with the reaction mixture maintained under stirring at 25 °C for 24 h. Oxidative polymerization was selected because it enables in situ polymer growth within the clay interlayer galleries and is more suitable for obtaining a powder-form hybrid nanocomposite than electrode-confined electrochemical polymerization. The oxidant solution was cooled before addition, and the polymerization temperature was kept constant in order to limit rapid bulk oxidation and to favor more homogeneous polymer growth within the clay galleries. The resulting black mass was collected by filtration, washed entirely with methanol and deionized water to remove residual monomer, oligomers, and oxidant, and lastly dried under vacuum at 60 °C for 24 h. The resultant material is denoted as PPy/Mag–Cu^2+^ nanocomposite.

### 2.4. Experimental Procedure for Adsorption Studies

Adsorption experiments were performed in batch mode to investigate the removal of methylene blue (MB) from aqueous solutions by the PPy/M–Cu^2+^ nanocomposite. For each run, 0.1 g of adsorbent was mixed with 50 mL of MB solution and agitated at 350 rpm at 14 °C. All adsorption experiments were conducted at this fixed temperature in order to ensure consistent evaluation of the selected operating parameters. At predetermined time intervals, the suspensions were centrifuged at 7000 rpm to separate the adsorbent from the solution. The kinetic experiments were carried out using the same batch setup, and samples collected at each contact time were likewise separated by centrifugation before UV-Vis analysis. The residual dye concentration was analyzed by UV-Vis spectrophotometry at λmax = 667 nm. The amount of dye adsorbed was determined from the difference between the initial and final dye concentrations. The main operating parameters investigated were contact time, pH, and initial dye concentration. All adsorption experiments were performed in triplicate, and the results are presented as mean ± standard deviation. Error bars in the adsorption plots represent the standard deviation of triplicate measurements. The pHpzc was determined by the pH drift method. Briefly, 50 mL of 0.01 M NaCl solution was adjusted to different initial pH values using 0.1 M HCl or 0.1 M NaOH, and then 0.1 g of PPy/M–Cu^2+^ nanocomposite was added to each solution. The suspensions were stirred for 24 h at room temperature, after which the final pH values were recorded. The pHpzc was taken as the point where the initial pH was equal to the final pH (ΔpH = 0). Contact time experiments were conducted over 0–120 min at pH 3.2 with an initial MB concentration of 40 ppm. The solution pH was varied from 3.2 to 11 using 1 M NaOH and 1 M HCl, whereas the initial concentration study was carried out in the range of 40–90 ppm. For the isotherm analysis, the equilibrium concentrations (Ce) obtained at different initial MB concentrations and the corresponding equilibrium adsorption capacities (qe) were used to construct the Langmuir and Freundlich plots. These experiments were designed to determine the optimal adsorption conditions and to evaluate the adsorption behavior of the synthesized nanocomposite. The adsorption capacity at equilibrium was calculated according to the following equation:

q_e_ = ((C_0_ − C_e_) × V)/m (1)

where C_0_ and C_e_ (mg/L) are the initial and equilibrium dye concentrations, q_e_ (mg g^−1^) is the equilibrium adsorption capacity, V (L) is the solution volume, and m (g) is the mass of adsorbent.

### 2.5. Characterizations

To ensure statistical robustness, reproducibility, and data dependability, the characterization experiments were carried out under consistent experimental conditions. In addition, the purified and sodium-activated clay samples used for characterization were prepared following the same protocol in order to verify the reproducibility of the pretreatment procedure. An explanation of the procedures used is provided below.

X-ray fluorescence, or XRF. Using a Philips PW1480 spectrometer equipped with UNIQUANT II software, version, for semi-quantitative elemental analysis (Philips, Eindhoven, The Netherlands), X-ray fluorescence spectroscopy was done to discover the elemental compositions. The evaluation was based on the relative intensities of the characteristic X-ray emission lines of the detected elements. This approach, which is based on the detection of characteristic X-ray emission lines, can successfully differentiate between the elemental compositions of pure and changed clays. The elemental values reported in Table 1 were obtained from this semi-quantitative evaluation. In the present work, the XRF results were considered semi-quantitative because they were used primarily for comparative assessment of compositional changes between samples rather than for absolute elemental quantification. Therefore, the reported values, particularly for light elements such as O, should be interpreted as approximate comparative data. The measurements were performed in circumstances that gave the best practicable signal detection and uniformity across all samples.

X-ray diffraction, or XRD. Powder X-ray diffraction patterns were acquired at room temperature using CuKα radiation (λ = 1.5406 Å) with a nickel filter using a Bruker CCD-Apex diffractometer (Bruker, Wissembourg, France) running at 40 kV and 40 mA. A step size of 0.05° and a scanning rate of 0.08° min^−1^ were utilized to obtain the data. These well-defined characteristics allow it to be viable to calculate accurate interlayer spacing, identify phases, and measure crystallinity throughout the modification and nanocomposite synthesis activities.

Fourier transform infrared spectroscopy (FT-IR). FT-IR spectra in the 4000–400 cm^−1^ area were acquired using a Bruker Alpha spectrometer. Samples were produced as KBr pellets under carefully regulated parameters to provide the highest signal quality, spectral accuracy, and consistency of band locations and intensities.

UV-visible spectroscopy (UV-Vis). A Hitachi U-3000 spectrophotometer (Hitachi, Tokyo, Japan) was utilized to collect electronic absorption spectra. For these experiments, samples were produced in dimethyl sulfoxide (DMSO) to create homogeneous solutions, assuring that the spectroscopic results correctly reflected the underlying optical properties of the materials.

Cyclic voltammetry (CV). The electrochemical behavior of the nanocomposite was studied after the removal of the polymeric component. After dissolving the nanocomposite in DMSO, the polymer was separated by filtering away the insoluble MMT-H^+^ component. To make thin films, the polymer solution was drop-cast onto graphite working electrodes and then progressively dried under infrared irradiation.

Using 1 M HClO_4_ as the supporting electrolyte, electrochemical measurements were carried out in a typical three-electrode cell. The design includes a platinum counter electrode, a graphite working electrode modified by polymers, and a reversible hydrogen electrode (RHE) operating as the reference electrode. Voltammograms were acquired at a scan rate of 50 mV·s^−1^, with particular regard to cell design and electrode conditioning, in order to give accurate and comparable data.

Every measurement for FT-IR, UV-Vis, XRD, and CV tests was done in triplicate on the same sample in order to establish repeatability and assess the robustness of the experimental data. The information presented here reveals the inherent qualities of the materials and is reflective of these independent measurements. SEM (scanning electron microscopy) surface morphology was investigated using a Hitachi S-6600L scanning electron microscope (Hitachi High-Tech Corporation, Tokyo, Japan) operating at a 20 kV accelerating voltage. To ensure representativeness, elemental mapping was performed over a typical area of 5.7 × 4.7 μm^2^. Since the concentrations match average values obtained from these mapped places, they give a realistic depiction of the elements’ distribution. Within the scope of the present work, the structural interpretation was primarily based on XRD, SEM, FT-IR, UV-Vis, and electrochemical characterization in correlation with the adsorption results.

Figure 1 was produced by the authors. Artificial intelligence software was not used to generate the figure as a whole; however, OpenAI’s ChatGPT (GPT-5.5 Thinking model, model ID: gpt-5.5) was used only in the editing of several visual elements included within the figure.

## 3. Results and Discussion

### 3.1. Physicochemical Properties of the Samples

#### 3.1.1. X-Ray Fluorescence (XRF) Analysis

The elemental composition of the pristine and modified maghnite samples, semi-quantitatively assessed by X-ray fluorescence spectrometry (XRF), suggests the successful and controlled stepwise chemical transformation. Because the XRF evaluation in this study was semi-quantitative, the values in Table 1 are interpreted mainly in terms of relative compositional changes among raw Mag, Mag–Na^+^, and Mag–Cu^2+^, rather than as absolute concentrations. In particular, the reported O content should be regarded as an approximate comparative value. The data, given in Table 1, reveal a distinct redistribution of elements following sodium activation, specifically changes in the relative abundance of Si and in the semi-quantitative reported O content, combined with the depletion of native exchangeable cations. This compositional shift is consistent with the effective replacement of the interlayer cation population by Na^+^ ions, a prerequisite for enhancing the clay’s swelling capacity and creating a more homogeneous and reactive interlayer environment, all while preserving the integrity of the aluminosilicate framework.

Subsequent Cu^2+^ exchange is corroborated by the evident presence of copper as a significant constituent in Table 1. The integration of Cu^2+^ occurs with a small decline in octahedral cations (e.g., Mg, Al), strongly hinting that copper ions are efficiently intercalated into the gallery spaces via ion exchange, rather than being present as surface-bound oxide/hydroxide phases. Given the semi-quantitative nature of the XRF analysis, the overall preservation of the major elemental profile, together with the XRD results, is consistent with the retention of the underlying 2:1 layer structure. From a functional aspect, this progressive tuning of the interlayer chemistry from a pure Na-form to a Cu^2+^-enriched one is purposely intended to provide multiple, synergistic adsorption sites, setting the stage for enhanced interaction with target adsorbates like methylene blue [32,33].

#### 3.1.2. X-Ray Diffraction (XRD) Analysis

**Figure 2:**
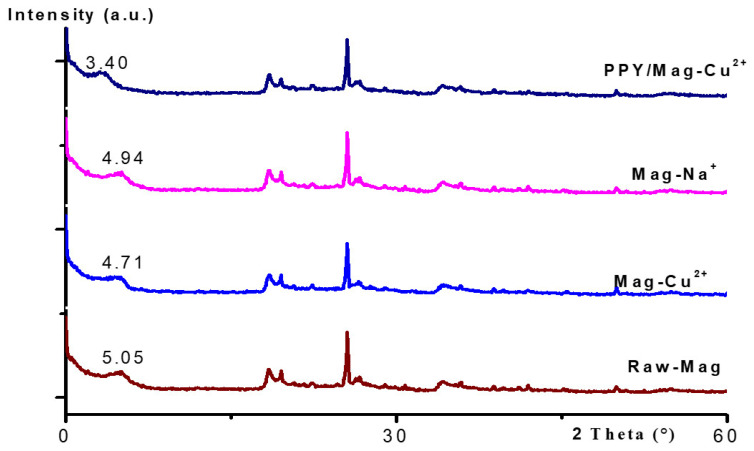
XRD patterns of raw maghnite, Maghnite–Na^+^, Maghnite–Cu^2+^, and PPy/Mag–Cu^2+^ nanocomposite, indicating the progressive expansion of the interlayer spacing (d_001_).

X-ray diffraction (XRD) was performed at every step of the modification technique to monitor the evolution of the interlayer spacing and confirm the structural changes. Figure 2 depicts the resulting diffractograms. At 2θ = 5.05°, which corresponds to a basal spacing (d_001_) of 17.49 Å, the raw maghnite diffractogram reveals the unique (d_001_) basal reflection. This value is typical of a well-ordered, hydrated smectite clay with a layered structure. After sodium activation, the (001) peak changes to a somewhat lower angle (2θ = 4.94°), which shows that the distance between the layers has increased to 17.89 Å. This tiny but observable rise is a direct result of more hydrated Na^+^ ions taking the place of the original interlayer cations, which shows that activation was effective. After the Cu^2+^ exchange, the basal reflection moves to 2θ = 4.71° (d_001_ = 18.77 Å), which indicates a bigger expansion. This steady expansion (17.49 → 17.89 → 18.77 Å) in Table 2 gives strong evidence that hydrated Cu^2+^ complexes are successfully intercalated inside the clay galleries. According to Bragg’s law (nλ = 2d sin θ), for a fixed X-ray wavelength, a shift in the basal reflection toward lower 2θ values corresponds to an increase in d-spacing. Therefore, the progressive displacement of the (001) peak from 5.05° to 3.40° directly confirms the increase in basal spacing during the successive modification steps.

The final PPy/Mag–Cu^2+^ nanocomposite displays the most pronounced structural change. In this case, the (001) reflection shifts markedly to 2θ = 3.40°, which implies that the d-spacing significantly increases to 25.95 Å. Based on Bragg’s law, this marked decrease in diffraction angle is fully consistent with the pronounced expansion of the interlayer distance from 17.49 Å for raw maghnite to 25.95 Å for the final nanocomposite. Ion exchange cannot explain this about 8.5 Å increase as compared to the original clay. Instead, it delivers convincing crystallographic proof of effective monomer intercalation, which is later followed by oxidative polymerization in the confined interlayer space [34,35]. This strategy is described in Figure 1. This degree of expansion suggests that the layers are more widely separated, supporting the formation of an intercalated nanostructure. This configuration is a very desirable design for adsorption applications.

From a practical point of view, this wider gallery is advantageous. It facilitates access to the internal surface area and makes it easier for large molecules such as methylene blue to diffuse to the active sites. The resulting nanostructure increases synergistic adsorption in the following ways:

- A larger electrostatic force between the negatively charged clay layers and the cationic dye.
- Specific coordination links with the Cu^2+^ intercalated centers.
- The aromatic rings and conjugated polypyrrole chains in methylene blue display complementary π–π interactions.

In conclusion, the measurement of the systematic change in the d001 spacing in Figure 2 gives unequivocal structural proof that the anticipated nanocomposite synthesis was successfully achieved. The crystallographic data clearly reveals that an extended, intercalated PPy/Mag–Cu^2+^ nanocomposite has been created. Its structure is suitable for adsorbing cationic pollutants at high speeds.

#### 3.1.3. FTIR Spectroscopic Analysis

**Figure 3:**
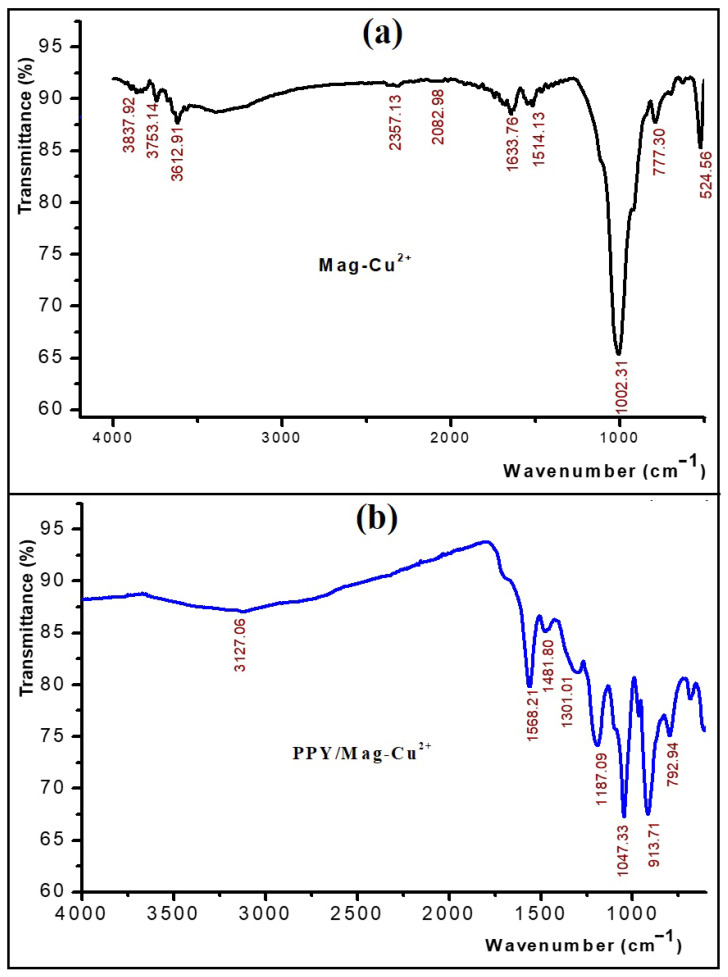
(**a**) FTIR spectra of modified clay (Mag–Cu^2+^); (**b**) FTIR spectrum of the PPy/Mag–Cu^2+^ nanocomposite.

As shown in Figure 3a, the FTIR spectra of maghnite-Cu^2+^-modified clay display the characteristic structural features of layered aluminosilicates, including the broad O–H stretching band at ~3400–3500 cm^−1^, the H–O–H bending vibration near ~1633 cm^−1^, and the strong Si–O–Si stretching band at ~1003 cm^−1^, confirming the preservation of the silicate framework after ion exchange. Minor changes in band intensity and morphology after Cu^2+^ exchange reflect adjustments in the interlayer hydration environment without rupture of the clay lattice. In contrast, Figure 3b reveals the emergence of new absorption bands characteristic of polypyrrole after in situ oxidative polymerization, notably at ~1568 cm^−1^ and ~1481 cm^−1^, corresponding to asymmetric and symmetric C=C stretching of the pyrrole ring, which originates from the five-membered heterocyclic unit of polypyrrole; at ~1301 cm^−1^, assigned to C–N stretching; and at ~1002 cm^−1^, associated with in-plane ring deformation, consistent with the characteristic vibrational features of polypyrrole derived from a five-membered pyrrolic ring [36,37]. The occurrence of these polymer-specific vibrations with the surviving silicate framework bands supports the effective creation of an organic–inorganic hybrid nanocomposite rather than a mere physical interaction. Moreover, the coexistence of clay-related and Ppy-related bands indicates that the layered aluminosilicate structure was retained after polymerization while new organic functionalities were successfully introduced into the hybrid material. Modest band broadening and slight modifications in the polymer region further suggest interfacial interactions between polypyrrole chains and the Cu^2+^-modified clay layers. From a functional standpoint, the material combines negatively charged silicate surfaces favoring electrostatic attraction of cationic methylene blue (MB), conjugated aromatic PPY domains enabling π–π stacking interactions, and surface hydroxyl and nitrogen-containing groups capable of hydrogen bonding, thereby providing multiple synergistic adsorption sites that enhance dye removal performance [38,39]. The main FTIR bands and their corresponding functional group assignments are summarized in Table 3.

#### 3.1.4. UV–Vis Analysis

**Figure 4:**
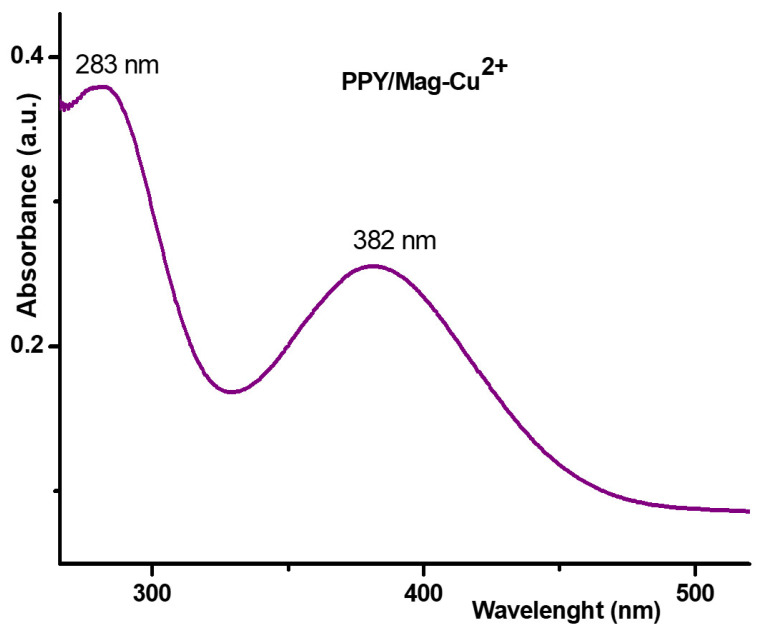
UV–Vis absorption spectra of the PPy/Mag–Cu^2+^ nanocomposite.

The PPy/Mag–Cu^2+^ nanocomposite displayed two strong absorption peaks in its UV–Vis spectra, which were located at roughly 283 nm and 382 nm (Figure 4). The development of a conjugated polypyrrole (PPy) framework is proven by the strong band at about 283 nm, which is connected to the π→π* electronic transition of the conjugated pyrrolic backbone. After oxidative polymerization, the polymer exists in a partially oxidized/doped form, as demonstrated by the second, bigger band at about 382 nm, which is attributed to oxidation-related electronic transitions in PPY (usually associated with polaronic states). These findings indicate that the electronic properties of the conjugated polypyrrole framework can be finely tuned through the degree of oxidation, enhancing its potential applications in electronic devices and sensors. Strong interfacial interactions between PPy chains and the Cu^2+^-modified clay galleries, where coordination/electrostatic coupling can stabilize charge carriers and promote delocalization along the polymer chain, are notably consistent with the presence and relative broadening of this near-UV/visible band. In summary, the dual-band profile validates the predicted nanocomposite structure by presenting spectroscopic evidence of effective PPy synthesis and electronic interactions within the hybrid architecture.

The occurrence of protracted conjugation and/or charge-carrier states typical of conducting polymer-based hybrids is further corroborated by the gradual drop in absorbance at longer wavelengths (up to ~520 nm).

#### 3.1.5. Microstructural Evolution of Mag–Cu^2+^ and PPy/Mag–Cu^2+^ Nanocomposite Observed by SEM

**Figure 5:**
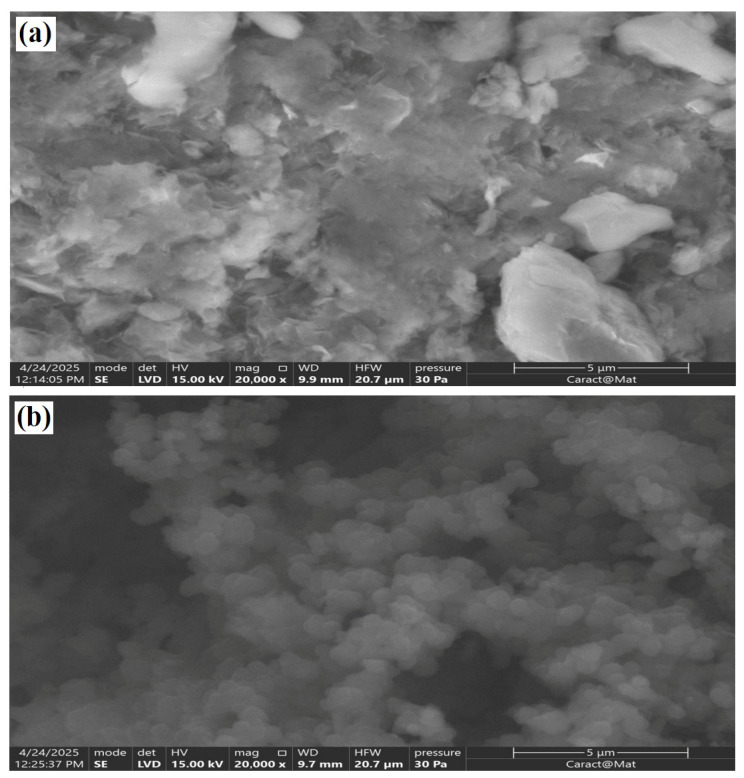
(**a**) SEM micrograph of Cu^2+^-modified maghnite (Mag–Cu^2+^); (**b**) SEM micrograph of the PPy/Mag–Cu^2+^ nanocomposite.

The SEM micrographs given in Figure 5 reveal clear morphological evidence of the structural modification occurring upon polymer insertion. As seen in Figure 5a, the Cu^2+^-modified maghnite displays the normal lamellar and plate-like morphology characteristic of layered smectite clays. The particles appear as stacked and partially aggregated sheets with irregular edges, reflecting the essential layered structure of montmorillonitic materials [40]. The observed morphology suggests the presence of relatively compact agglomerates formed by overlapping clay platelets, which is consistent with the natural tendency of layered particles to restack after drying. The surface texture remains mostly compact, although modest interparticle spacing and surface roughness may be identified, probably originating from interlayer expansion during cation exchange. Importantly, no identifiable crystalline Cu-containing secondary phases are found, demonstrating that Cu^2+^ is primarily absorbed via ion exchange rather than producing discrete oxide or hydroxide particles.

In contrast, Figure 5b depicts a substantially changed surface morphology after in situ polymerization of pyrrole. The nanocomposite exhibits a more heterogeneous and roughened surface, defined by a granular and connected network structure. The lamellar properties of the clay become partially covered by a continuous polymer coating, indicating successful growth of polypyrrole on and potentially within the clay layers. The enhanced surface irregularity and the building of a porous-like architecture indicate greater surface accessibility and the production of more adsorption-active sites. The absence of big polymer clumps further confirms a rather uniform distribution of PPy throughout the clay matrix rather than just physical mixing. Compared with Mag–Cu^2+^, the nanocomposite also shows a less compact mode of aggregation, with interconnected domains that appear more open and spatially distributed across the surface. This change suggests that polymer formation between and around the clay platelets reduces dense restacking and promotes a more accessible agglomerated structure. From a functional aspect, this morphological alteration is especially relevant to methylene blue (MB) adsorption. The shift from compact clay platelets to a rough, polymer-modified hybrid structure is projected to boost surface area, promote dye dispersion, and provide diverse interaction domains. The clay layers provide negatively charged adsorption sites, whereas the polypyrrole phase introduces π-conjugated domains capable of π–π interactions with the aromatic rings of methylene blue. The synergistic combination of expanded interlayer spacing, increased surface heterogeneity, and polymer functionalization is expected to improve adsorption performance by enhancing accessibility and promoting multiple interaction pathways [41,42,43]. In general, the SEM analysis backs up the successful creation of a structurally integrated organic-inorganic nanocomposite and shows morphological proof that it works better for removing methylene blue. Although no numerical particle size distribution was extracted from the SEM images in the present study, the observed micrographs clearly indicate a transition from compact platelet agglomerates to a more open and heterogeneous hybrid morphology after polymer incorporation.

#### 3.1.6. Analysis of Cyclic Voltammetry

**Figure 6:**
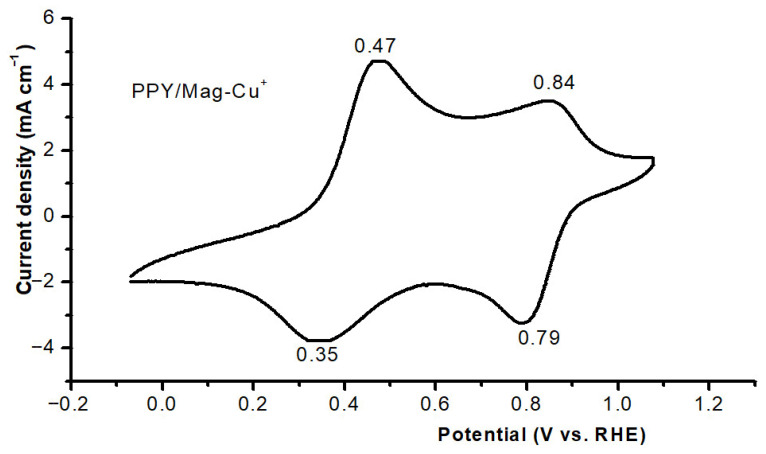
Cyclic voltammogram of polypyrrole isolated from the PPy/Maghnite–Cu^2+^ nanocomposite recorded in 1 M HClO_4_ at a scan rate of 50 mV s^−1^.

In the cyclic voltammogram of the PPy/Mag–Cu^2+^, a typical conducting-polymer response with two distinct redox couples can be observed. As shown in Figure 6, the cyclic voltammogram exhibits two distinct redox couples characteristic of conducting polypyrrole. This indicates changes in the oxidation state of the polypyrrole structure. The first anodic peak at 0.47 V and the second cathodic peak at 0.35 V are both attributed to the initial p-doping phase of PPy. This phase involves the formation of polaronic charge carriers along the conjugated chain and the uptake of counter-ions from the electrolyte to keep the charge neutral. The second redox pair appears at a higher potential (anodic 0.84 V, cathodic 0.79 V), which implies a more advanced oxidation state. The corresponding oxidation and reduction peak parameters are summarized in Table 4. This is generally associated with the transition to bipolaronic states and/or increased doping in the polymer matrix. The small peak separations between the peaks (ΔEp = 0.12 V for the first pair and 0.05 V for the second) show that the charge transfer is fast and that the redox activity is essentially reversible. This is especially relevant for polymeric films, as bulk ion transport often broadens the peaks and increases polarization. The fact that there are two different couples shows that there are multiple redox environments (surface vs. bulk sites, or areas influenced by the inorganic clay/Cu^2+^ environment). This is consistent with a nanocomposite-derived PPy structure. The voltammetric signature reveals that the PPy component is still extremely active electrically, which is crucial for dye removal [44,45]. The redox-switchable charge density of PPY may make electrostatic interactions with cationic adsorbates (like methylene blue) stronger. The π-conjugated backbone may also assist with extra π–π interactions, in agreement with previous reports on polypyrrole-based nanocomposites.

### 3.2. Adsorption Study of MB on Adsorbents

#### 3.2.1. Effect of Contact Time

**Figure 7:**
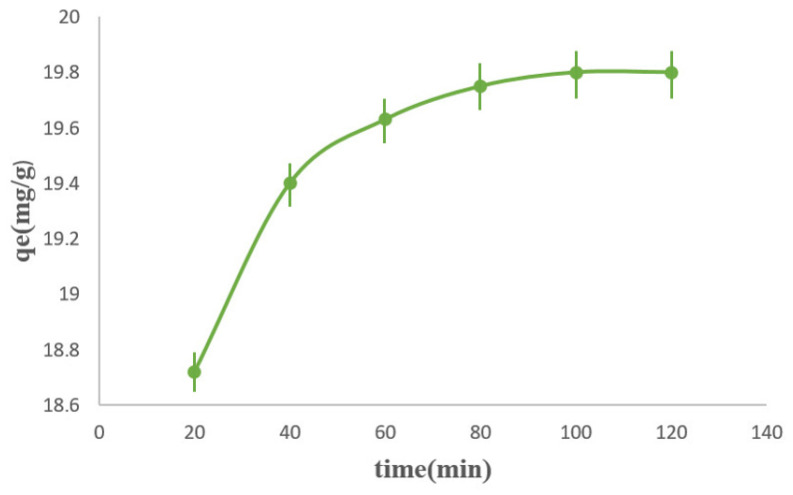
The effect of contact time on MB adsorption onto PPy/M–Cu^2+^.

The effect of contact time on the adsorption of methylene blue (MB) onto the Ppy/M–Cu^2+^ nanocomposite was investigated over the range of 0–120 min. As shown in Figure 7, the adsorption capacity increased rapidly during the first stage of the process, then more gradually until equilibrium was reached after approximately 80 min. Beyond this contact time, no significant increase in adsorption was observed, indicating that the available active sites on the adsorbent surface had become nearly saturated. Under these experimental conditions, the equilibrium adsorption capacity (q_e_,exp) was about 19.8 mg g^−1^. This rapid initial uptake may be attributed to the large number of vacant adsorption sites initially available on the surface of the nanocomposite, which facilitated the fixation of MB molecules; as contact time increased, the adsorption rate progressively decreased due to the gradual occupation of these active sites and the reduction in the concentration gradient between the bulk solution and the adsorbent surface. Such a two-step adsorption profile, involving a fast surface adsorption step followed by a slower approach to equilibrium, has been widely reported for MB adsorption on clay-based and composite materials [46,47,48]. The slower second stage also suggests that, in addition to surface adsorption, mass transfer limitations may contribute during the approach to equilibrium. In the present study, the equilibrium time of 80 min suggests a relatively good affinity between the PPy/M–Cu^2+^ nanocomposite and the cationic dye. This interpretation is consistent with the kinetic analysis, in which the pseudo-second-order model provided the best fit to the experimental data, indicating that the overall adsorption rate was strongly dependent on the availability of adsorption sites and surface interactions between the adsorbent and adsorbate. Compared with recent literature, the equilibrium time obtained in this work is close to that reported for natural muscovite–kaolinite clay, for which adsorption equilibrium was reached after about 60 min, and is also in agreement with studies on natural and purified clays, where rapid initial adsorption followed by progressive stabilization was observed. A similar behavior was reported for a clay/carbon composite, where the adsorption kinetics also followed the pseudo-second-order model and the equilibrium process was well described by the Langmuir model. In contrast, some recently developed biosorbents exhibited even faster adsorption, with equilibrium reached within 30 min, highlighting that the contact time strongly depends on the textural and chemical properties of the adsorbent. Therefore, the contact time profile obtained in the present study confirms that the synthesized PPy/M–Cu^2+^ nanocomposite possesses a satisfactory adsorption performance toward methylene blue and behaves similarly to several efficient adsorbents reported in recent studies [49,50].

#### 3.2.2. Effect of pH

**Figure 8:**
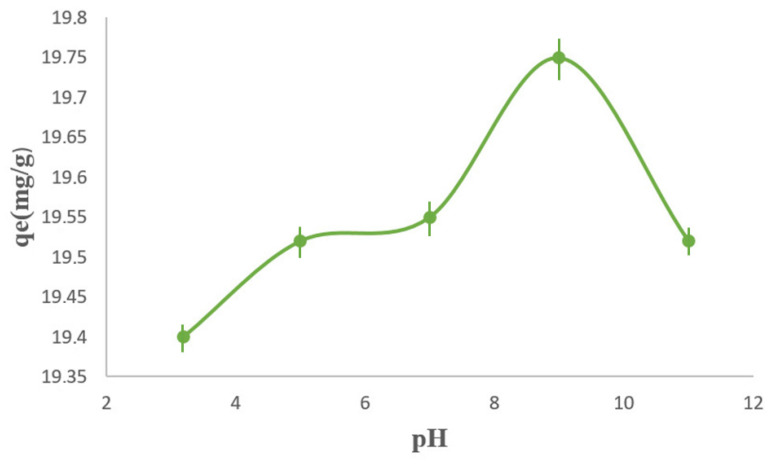
Effect of pH on MB adsorption onto PPy/M–Cu^2+^.

The effect of solution pH on the adsorption of methylene blue (MB) onto the PPy/M–Cu^2+^ nanocomposite was investigated over the pH range 3.2–11. According to Figure 8, the adsorption capacity increased progressively with increasing pH, reaching a maximum value of about 19.75 mg g^−1^ at pH 9, then showing a slight decline at higher pH values. This behavior clearly indicates that the pH of the medium plays a key role in controlling the adsorption performance of the synthesized material. The observed trend can be explained by considering both the ionic form of methylene blue and the surface charge of the adsorbent. Since methylene blue is a cationic dye, its adsorption is strongly influenced by electrostatic interactions with the surface of the adsorbent. In the present study, the pH at the point of zero charge (pHpzc) of the PPy/M–Cu^2+^ nanocomposite was found to be 3.4. This value is important because it defines the pH at which the net surface charge changes and therefore provides a direct basis for interpreting the pH-dependent adsorption behavior. Therefore, at pH values higher than pHpzc, the adsorbent surface becomes predominantly negatively charged, which enhances the electrostatic attraction between the surface and the positively charged MB molecules, leading to higher adsorption capacities. In contrast, at acidic pH values below pHpzc, the surface is more positively charged, resulting in electrostatic repulsion with MB cations and, consequently, lower adsorption efficiency. Similar pH-dependent behavior has been widely reported for MB adsorption on clay-based, mineral, and composite adsorbents, where adsorption generally improves under neutral to alkaline conditions because of the increasing density of negatively charged surface sites. The increase in adsorption up to pH 9 is therefore consistent with the progressive deprotonation of surface functional groups and the strengthening of attractive interactions between the dye and the adsorbent surface. The slight decrease observed beyond the optimum pH may be related to changes in the adsorption environment, including possible competition with excess hydroxyl ions or modification of the interfacial interactions at strongly alkaline conditions. A comparable trend was reported for natural muscovite–kaolinite clay, where MB adsorption increased with pH and reached its optimum under alkaline conditions. Likewise, adsorption studies on natural and purified clays showed that higher pH values favor the removal of methylene blue because the clay surface becomes more negatively charged and more accessible to cationic dye molecules. Recent work on ceramic clay-based filters also confirmed that the adsorption of MB is strongly governed by pH, with improved uptake in alkaline media due to favorable electrostatic attraction. In addition, a recent review on MB adsorption emphasized that the relationship between solution pH and pHpzc is one of the main factors governing adsorption efficiency for cationic dyes, particularly when the mechanism is dominated by surface charge effects. Therefore, the results obtained in the present study confirm that pH 9 represents the optimum condition for MB adsorption onto the PPy/M–Cu^2+^ nanocomposite and demonstrate that electrostatic attraction is one of the main driving forces of the adsorption process.

#### 3.2.3. Effect of Initial Dye Concentration

**Figure 9:**
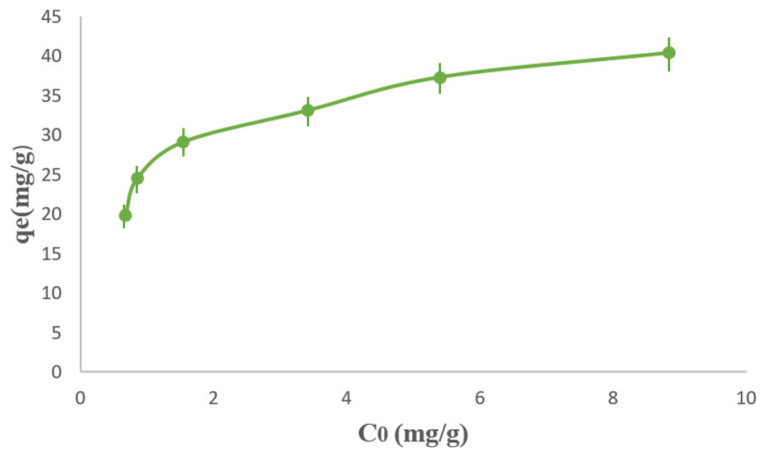
Effect of Initial MB Concentration on Adsorption Capacity onto PPy/Mag–Cu^2+^.

As shown in Figure 9, the effect of the initial methylene blue (MB) concentration on the adsorption performance of the PPy/M–Cu^2+^ nanocomposite was investigated under the previously optimized operating conditions. The results showed that the adsorption capacity increased with increasing initial dye concentration, which can be explained by the rise in the concentration gradient between the bulk solution and the adsorbent surface. At low initial concentrations, the number of MB molecules in solution remains relatively limited compared with the available active sites, whereas at higher concentrations the driving force for mass transfer becomes stronger, leading to a greater amount of dye adsorbed per unit mass of adsorbent. This behavior, illustrated in Figure 9, is commonly observed in adsorption systems and indicates that the initial concentration is an important parameter controlling the adsorption capacity of the material. The increase in adsorption capacity with increasing MB concentration can also be attributed to the enhanced probability of collision between dye molecules and the active sites of the adsorbent surface. However, although the adsorption capacity increases, the removal efficiency may not increase proportionally at high concentrations because the available adsorption sites progressively approach saturation. Therefore, the observed trend shown in Figure 9 suggests that the PPy/M–Cu^2+^ nanocomposite possesses an appreciable affinity for MB over the investigated concentration range, while still being limited by the finite number of accessible adsorption sites. Similar behavior has been reported in recent studies on methylene blue adsorption by natural muscovite–kaolinite clay, where the adsorption capacity increased with the initial dye concentration due to an enhanced mass transfer driving force. A comparable trend was also observed for natural and purified clays, in which higher initial MB concentrations led to higher adsorption capacities until the progressive saturation of surface sites became significant. Likewise, adsorption studies using clay/carbon composites confirmed that increasing the initial dye concentration promotes uptake at equilibrium, although the adsorption process ultimately becomes limited by site availability and pore accessibility. More broadly, recent reviews have emphasized that the initial dye concentration is one of the main experimental factors governing adsorption performance, because it directly affects both the mass transfer gradient and the equilibrium distribution of dye molecules between the solution and the adsorbent surface. Although adsorbent dosage was not examined as an independent variable in the present study, the fixed amount of adsorbent used throughout the experiments provided a consistent basis for evaluating the effects of the selected operating parameters. Overall, the results presented in Figure 9 confirm that increasing the initial MB concentration enhances the adsorption capacity of the PPy/Mag–Cu^2+^ nanocomposite, while the final adsorption level remains controlled by the number and accessibility of the active sites available on the adsorbent surface.

#### 3.2.4. Adsorption Isotherms

To better understand the adsorption behavior of methylene blue (MB) onto the synthesized PPy/Mag–Cu^2+^ nanocomposite, the experimental equilibrium data were analyzed using the Langmuir and Freundlich isotherm models, which are commonly employed to describe dye adsorption mechanisms and the interaction between adsorbate molecules and the adsorbent surface. These models provide valuable information about the nature of the adsorption surface, the distribution of active sites, and the possible occurrence of monolayer or multilayer adsorption. The Langmuir model assumes monolayer adsorption on a homogeneous surface containing a finite number of identical active sites and is expressed as:

(2) $$q_{e}=\frac{Q_{0}K_{L}\;C_{e}}{1+K_{L}\;C_{e}}$$

whereas the Freundlich model describes adsorption on a heterogeneous surface with sites of different energies and is given by:

(3) $$q_{e}=K_{F}C_{e}^{1/n}$$

The linearized forms of these models used for the determination of the isotherm parameters are written as:

(4) $$\frac{\mathrm{Ce}}{\mathrm{qe}}=\frac{\mathrm{Ce}}{Q_{0}}+\;\frac{1}{K_{L\;}Q_{0}}$$

(5) $$\;\ln q_{e}=\ln K_{F}+\frac{1}{n}\ln C_{e}$$

**Figure 10:**
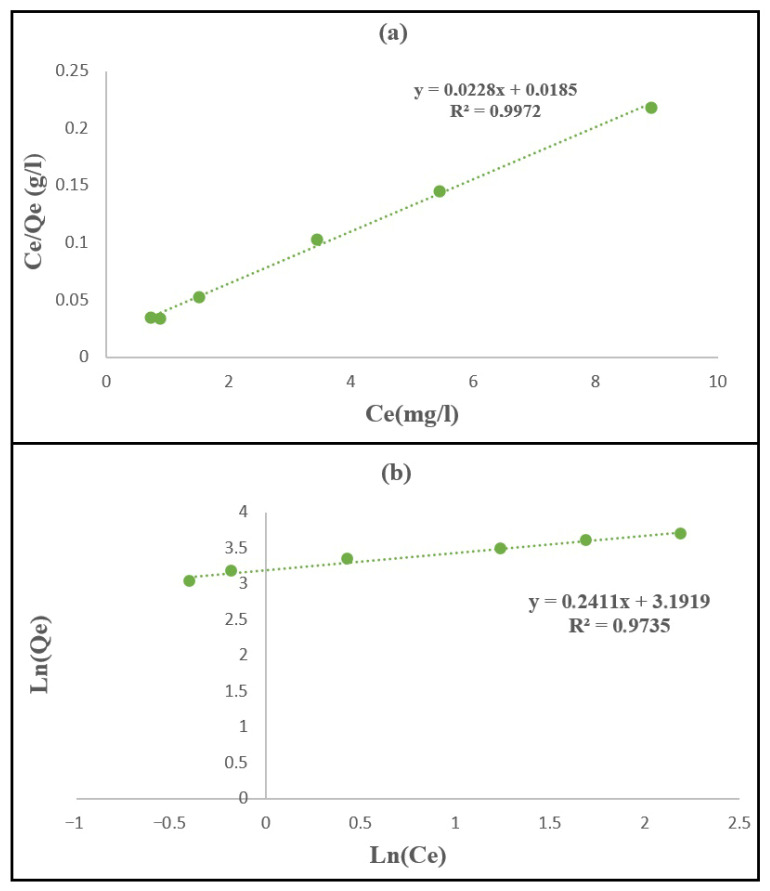
Linearized adsorption isotherm plots: (**a**) Langmuir model and (**b**) Freundlich model in the natural logarithmic form for MB adsorption onto PPy/Mag–Cu^2+^ nanocomposite.

In the present study, the Freundlich linearization was expressed in its natural logarithmic form, consistent with Figure 10b. The linearized plots shown in Figure 10 were constructed from the experimental equilibrium C_e_ and q_e_ values obtained from the adsorption experiments. To better understand the equilibrium behavior of methylene blue (MB) adsorption onto the synthesized PPy/Mag–Cu^2+^ nanocomposite, the experimental data were analyzed using the Langmuir and Freundlich isotherm models. As illustrated in Figure 10 and summarized in Table 5, the Langmuir model provided a significantly better fit to the experimental equilibrium data than the Freundlich model, with a correlation coefficient of R^2^ = 0.997 compared with R^2^ = 0.9735, respectively. In addition, the maximum monolayer adsorption capacity obtained from the Langmuir model (qmax) was 43.66 mg g^−1^. This value is distinct from the experimental equilibrium adsorption capacity (q_e_,exp) obtained in the contact time experiments (19.8 mg g^−1^), since qmax represents the theoretical maximum monolayer capacity estimated from isotherm modeling, whereas q_e_,exp reflects the adsorption capacity measured under a specific set of experimental conditions. These findings indicate that MB adsorption onto the PPy/Mag–Cu^2+^ nanocomposite is predominantly described by monolayer adsorption on a relatively homogeneous surface, where the active sites are assumed to be energetically equivalent and each site retains one dye molecule without significant interaction between adsorbed species [51,52]. The lower correlation obtained with the Freundlich model suggests that surface heterogeneity and multilayer adsorption are less representative of the present adsorption system. From a mechanistic point of view, this adsorption behavior is consistent with the presence of accessible and functionally cooperative sites on the hybrid surface. The negatively charged clay layers favor electrostatic attraction with cationic MB molecules, while the polypyrrole phase contributes π-conjugated domains that can interact with the aromatic structure of MB through π–π interactions. In addition, the Cu^2+^-modified clay environment and the nitrogen-containing groups of the composite may contribute to specific surface interactions, thereby strengthening dye fixation on the adsorbent. Similar behavior has been reported in several recent studies on methylene blue adsorption, where the Langmuir model provided the best description of equilibrium data for clay-based and composite adsorbents. For instance, a recent study on natural muscovite–kaolinite clay showed that the adsorption of methylene blue was better fitted by the Langmuir isotherm, with a maximum adsorption capacity of 70.93 mg g^−1^, confirming the predominance of homogeneous adsorption sites and monolayer coverage. Likewise, a clay/carbon composite studied in 2024 also followed the Langmuir model, with a reported maximum capacity of 29.54 mg g^−1^, which is lower than that obtained in the present work, suggesting that the PPy/Mag–Cu^2+^ nanocomposite exhibits comparatively better adsorption performance under the investigated conditions. In the same context, activated bentonite clay was also reported to exhibit efficient methylene blue adsorption with equilibrium behavior consistent with Langmuir assumptions, further supporting the applicability of this model to mineral-based adsorbents. Therefore, the better agreement of the present experimental data with the Langmuir model, as shown in Figure 10 and Table 5, confirms that the surface of the PPy/Mag–Cu^2+^ nanocomposite provides a relatively uniform distribution of adsorption sites toward methylene blue molecules. Moreover, the value of qmax = 43.66 mg g^−1^ demonstrates that the synthesized material has a satisfactory adsorption capacity, higher than some recently reported clay/carbon systems but lower than certain optimized clay adsorbents, which may be attributed to differences in textural properties, surface area, pore structure, and the density of functional groups involved in dye binding. Overall, the equilibrium results suggest that the adsorption mechanism is mainly governed by monolayer uptake of MB on active sites where electrostatic attraction is dominant, with additional contributions from π–π interactions and specific surface interactions within the PPy/Mag–Cu^2+^ hybrid structure. Overall, these results indicate that the adsorption of methylene blue onto PPy/Mag–Cu^2+^ is mainly governed by a Langmuir-type isotherm, reflecting monolayer adsorption on relatively homogeneous active sites and confirming the promising potential of this nanocomposite for the removal of cationic dyes from aqueous media. It should also be noted that the present adsorption evaluation was performed in single-solute model solutions under controlled batch conditions, so the reported performance reflects the intrinsic affinity of the nanocomposite toward methylene blue in the absence of competing ions.

#### 3.2.5. Kinetic Studies

**Figure 11:**
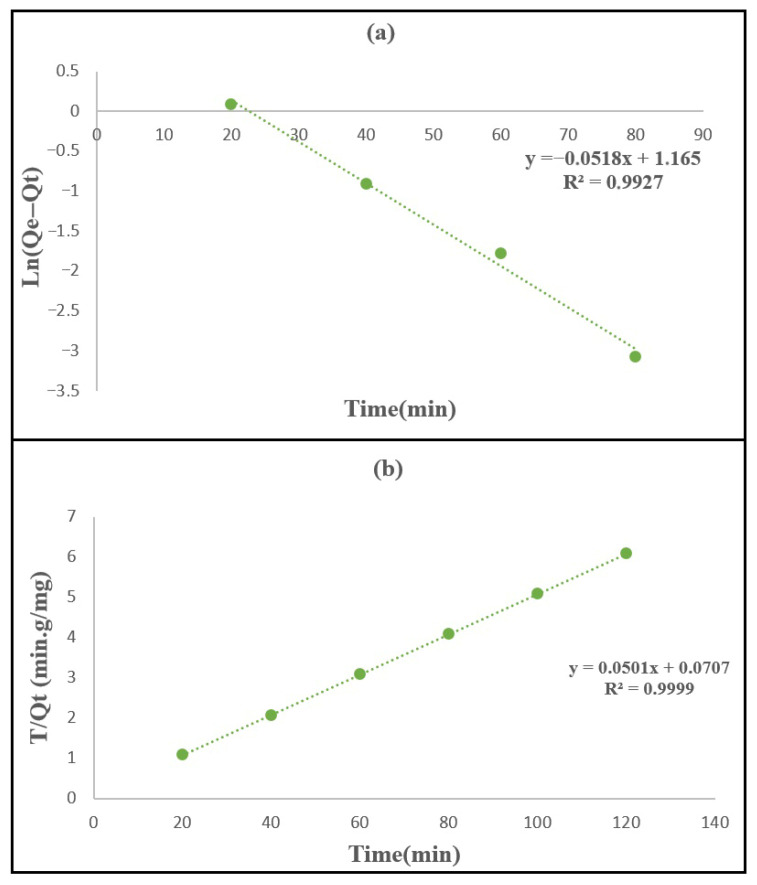
Linearized kinetic model plots: (**a**) pseudo-first-order and (**b**) pseudo-second-order for MB adsorption onto PPy/M–Cu^2+^ nanocomposite.

The adsorption kinetics of methylene blue (MB) onto the synthesized PPy/M–Cu^2+^ nanocomposite were evaluated using the pseudo-first-order (PFO) and pseudo-second-order (PSO) kinetic models in order to better understand the rate and mechanism of the adsorption process. The kinetic data were obtained from the same batch adsorption procedure described in Section 2.4, with centrifugation used at each sampling time prior to concentration analysis. As presented in Figure 11 and summarized in Table 5, the kinetic results showed that the pseudo-second-order model provided a better fit to the experimental data than the pseudo-first-order model, as confirmed by the higher correlation coefficient (R^2^ = 0.9999 for PSO versus R^2^ = 0.82 for PFO). This excellent agreement indicates that the adsorption of MB onto PPy/M–Cu^2+^ is mainly governed by surface-related interactions and by the progressive occupation of the available active sites on the adsorbent. Such behavior has been widely reported for methylene blue adsorption on clay-based and composite materials, where the pseudo-second-order model generally gives the best description of the kinetic data and is associated with strong adsorbate adsorbent interactions. The experimental adsorption profile also showed a rapid uptake during the first stage of contact, followed by a slower increase until equilibrium was reached after approximately 80 min, with an experimental adsorption capacity of about 19.8 mg g^−1^. This kinetic behavior can be explained by the large number of vacant active sites initially available on the surface of the nanocomposite, which promotes fast adsorption of MB molecules at the beginning of the process; as contact time increases, these sites become progressively occupied, and the adsorption rate decreases until equilibrium is established. Therefore, the fast initial stage may be attributed to external surface adsorption, while the slower stage reflects the gradual reduction in the number of free adsorption sites and possible diffusion resistance during the final approach to equilibrium. This two-step kinetic profile is also compatible with a combined adsorption mechanism in which MB molecules are first rapidly attracted to the external negatively charged and polymer-modified surface, followed by slower diffusion and fixation at the more accessible internal and interfacial sites of the composite. In this stage, electrostatic attraction, π–π interactions between MB and polypyrrole, and specific interactions involving Cu^2+^-modified sites may operate simultaneously. Similar two-step kinetic profiles have been reported in recent studies on methylene blue adsorption using natural muscovite–kaolinite clay, natural and purified clays, and clay/carbon composites, all of which also showed better agreement with the pseudo-second-order model [53,54,55]. Overall, the kinetic results shown in Figure 11 and Table 6 clearly indicate that methylene blue adsorption onto the PPy/Mag-Cu^2+^ nanocomposite follows a pseudo-second-order-controlled mechanism, with rapid adsorption in the early stage and equilibrium reached within a relatively short contact time. The very high R^2^ value obtained for the PSO model confirms the strong affinity between the cationic dye and the active sites of the synthesized material. Although diffusion phenomena may contribute during the later stage of adsorption, they do not appear to be the sole rate-limiting step, which is consistent with recent literature on MB adsorption highlighting that clay-based and composite adsorbents often exhibit pseudo-second-order kinetics because of favorable electrostatic attraction, surface complexation, and progressive saturation of adsorption sites. Accordingly, the present results support a mechanism dominated by surface adsorption with a secondary contribution of mass transfer effects during the later stage, while a more detailed separation of intraparticle diffusion, film diffusion, and other kinetic contributions would require additional model analysis. Taken together, the kinetic and equilibrium results suggest that MB removal by PPy/Mag–Cu^2+^ proceeds through a cooperative mechanism dominated by surface adsorption on active sites, rather than by diffusion alone.

## 4. Other Adsorbent-Based Studies

In order to better position the performance of the synthesized PPy/M–Cu^2+^ nanocomposite, the adsorption results obtained in the present study were compared with those reported for other adsorbents used for methylene blue (MB) removal from aqueous solutions. As shown in Table 7, the maximum adsorption capacities reported in the literature vary over a wide range depending on the physicochemical properties of the adsorbent, including its surface area, porosity, surface functionality, and degree of modification. In this context, the Langmuir maximum adsorption capacity obtained in the present work for PPy/M–Cu^2+^ (43.66 mg·g^−1^) indicates a satisfactory adsorption performance and places the synthesized material within an intermediate and competitive range among recently reported low-cost and mineral-based adsorbents. The obtained value is higher than those reported for some adsorbents such as clay/carbon composite, activated bentonite clay, natural Saudi zeolite, and phosphogypsum-tailings-derived zeolite, which confirms the good affinity of the prepared nanocomposite toward methylene blue. However, the adsorption capacity remains lower than that reported for natural muscovite–kaolinite clay, suggesting that the adsorption performance still depends strongly on the accessibility and density of active sites. This difference may be attributed to variations in surface homogeneity, pore structure, and the number of functional groups involved in dye binding. More specifically, the adsorption capacity of 43.66 mg·g^−1^ obtained in the present study is higher than the values reported for clay/carbon composite, activated bentonite clay, natural Saudi zeolite, and phosphogypsum-tailings-derived zeolite but lower than that of natural muscovite–kaolinite clay. This comparison suggests that the PPy/M–Cu^2+^ nanocomposite provides a competitive adsorption performance among recently reported mineral-based adsorbents, especially when considering its hybrid interlayer-expanded structure and the favorable agreement of the equilibrium data with the Langmuir model. Nevertheless, the relatively good capacity obtained in this work, together with the favorable Langmuir isotherm fit and the excellent agreement with the pseudo-second-order kinetic model, confirms that the PPy/M–Cu^2+^ nanocomposite is a promising adsorbent for the removal of cationic dyes from aqueous media. Furthermore, these results suggest that additional surface or structural modification could further improve its adsorption efficiency and make it comparable to the best-performing adsorbents described in the recent literature. For clarity, Table 7 summarizes the main equilibrium conditions or reported comparison conditions available in the cited studies, rather than limiting the comparison only to pH and temperature.

## 5. Conclusions

In this study, an interlayer-expanded PPy/Mag–Cu^2+^ nanocomposite was successfully synthesized through purification and sodium activation of raw maghnite, followed by Cu^2+^ ion exchange and in situ oxidative polymerization of pyrrole. The combined XRD, FTIR, UV–Vis, SEM, and cyclic voltammetry analyses confirmed the successful formation of the targeted organic–inorganic hybrid material. In particular, XRD results demonstrated a progressive increase in basal spacing from raw maghnite to the final nanocomposite, confirming both Cu^2+^ intercalation and pyrrole polymerization within the clay galleries. The adsorption experiments showed that PPy/Mag–Cu^2+^ is an effective adsorbent for methylene blue removal from aqueous solution, with equilibrium reached after approximately 80 min and optimal performance obtained at pH 9. The equilibrium data were best fitted by the Langmuir isotherm model, with a maximum monolayer adsorption capacity of 43.66 mg g^−1^, indicating adsorption on relatively homogeneous active sites. In addition, the adsorption kinetics were better described by the pseudo-second-order model, suggesting that the uptake process is mainly governed by surface interactions and progressive occupation of the available adsorption sites. Overall, the adsorption performance of PPy/Mag–Cu^2+^ can be attributed to the synergistic combination of the expanded interlayer structure of maghnite, Cu^2+^ coordination sites, negatively charged clay surfaces, and the π-conjugated redox-active polypyrrole network, which together promote electrostatic attraction, π–π interactions, and surface complexation.

Beyond its effectiveness for dye removal, this hybrid material also shows potential as a low-cost multifunctional platform for advanced water treatment applications. Its interfacial characteristics may be relevant for the development of pollutant capture materials and engineered surfaces designed to reduce undesired deposition or fouling in aqueous environments. Future work should therefore focus on regeneration and reusability studies to better assess the practical applicability and economic feasibility of the adsorbent, as well as thermodynamic analysis, further surface optimization, detailed textural characterization such as BET surface area, pore volume, and pore size distribution measurements, quantitative SEM image analysis of particle size distribution and agglomeration behavior, and more comprehensive kinetic modeling, including diffusion-based and higher-order models, in order to further clarify the adsorption mechanism, improve adsorption capacity, and extend the applicability of this material to other classes of organic pollutants and broader environmental applications.

## Figures and Tables

**Table 1:** Semi-quantitative elemental composition (wt%) of raw maghnite, Na^+^-exchanged maghnite (Mag Na^+^), and Cu^2+^-exchanged maghnite (Mag Cu^2+^).

Elemental Compositions (wt%)	Raw Mag	Mag Na^+^	Mag Cu^2+^
O	36.7	44.3	39.4
Si	23.2	30.3	27.5
Al	9.36	8.35	6.85
Mg	1.49	1.4	0.861
Fe	1.44	1.22	1.14
K	0.774	1.09	1.08
Ti	0.0779	0.0935	0.168
Mn	0.0558	0	0
Rb	0.0229	0.011	0.0213
Sr	0.0148	0	0
Zn	0.0134	0	0
Zr	0.00715	0.00653	0.0107
Ca	0.00715	0.00715	0.00715
Nb	0.0061		0.00649
P	0.00436	0.00436	0.00436
Y	0.00277	0	0
Cl	0	0.0544	0
Cu	0	0	1.99

**Table 2:** Basal reflection (001) location and computed d-spacing of raw maghnite, modified maghnite, and PPy/Mag–Cu^2+^ nanocomposite.

Sample	2θ (°)	θ (°)	d_001_ (Å)	d_001_ (nm)
Raw Maghnite	5.05	2.525	17.49	1.749
Maghnite–Na^+^	4.94	2.47	17.89	1.789
Maghnite–Cu^2+^	4.71	2.355	18.77	1.877
PPy/Maghnite–Cu^2+^ Nanocomposite	3.40	1.70	25.95	2.595

**Table 3:** Main FTIR bands of Mag–Cu^2+^ and PPy/Mag–Cu^2+^ and their assignments.

Sample	Band Position (cm^−1^)	Assignment
Mag–Cu^2+^	~3400–3500	O–H stretching
Mag–Cu^2+^	~1633	H–O–H bending
Mag–Cu^2+^	~1003	Si–O–Si stretching
PPy/Mag–Cu^2+^	~1568	Asymmetric C=C stretching of pyrrole ring
PPy/Mag–Cu^2+^	~1481	Symmetric C=C stretching of pyrrole ring
PPy/Mag–Cu^2+^	~1301	C–N stretching
PPy/Mag–Cu^2+^	~1002	In plane ring deformation

**Table 4:** XRD parameters (2θ, θ, and d001) of raw maghnite, Maghnite–Na^+^, Maghnite–Cu^2+^, and PPy/Maghnite–Cu^2+^ nanocomposite.

Sample	2θ (°)	θ (°)	d_001_ (Å)	d_001_ (nm)
Raw Maghnite	5.05	2.525	17.49	1.749
Maghnite–Na^+^	4.94	2.47	17.89	1.789
Maghnite–Cu^2+^	4.71	2.355	18.77	1.877
PPy/Maghnite–Cu^2+^ Nanocomposite	3.40	1.70	25.95	2.595

**Table 5:** Isotherm parameters for MB adsorption onto PPy/M–Cu^2+^ nanocomposite.

Model	Parameter	Value
Langmuir Model	Qmax (mg g^−1^)	43.66
	b (L mg^−1^)	1.21
	R^2^	0.997
Freundlich Model	Kf	24.34
	1/n	0.2411
	R^2^	0.9735

**Table 6:** Kinetic parameters of pseudo-first-order and pseudo-second-order models for MB adsorption onto PPy/Mag–Cu^2+^ nanocomposite.

Model	Parameter	Value
Pseudo-first-order $\ln\left(q_{e}-q_{t}\right)=\ln\left(q_{e}\right)-k_{1}t$	k_1_ (min^−1^)	0.018
	q_e_ (mg·g^−1^)	19.8
	R^2^	0.82
Pseudo-second-order $\frac{t}{q_{t}}=\frac{1}{{k_{2}q_{e}}^{2}}+\frac{t}{q_{e}}$	k_2_ (g mg^−1^ min^−1^)	0.044
	q_e_ (mg g^−1^)	3.00
	R^2^	0.9999

**Table 7:** Comparison of maximum adsorption capacities (qmax) for methylene blue on PPy/M–Cu^2+^ and other adsorbents.

No.	Adsorbent	Adsorbate	qmax (mg·g^−1^)	Reported Equilibrium Conditions/Remarks	Reference
1	Ppy/M-Cu^2+^ nanocomposite—this work	MB	43.66	pH 9; ~287 K; equilibrium reached at ~80 min	Present study
2	Natural muscovite–kaolinite clay	MB	70.93	alkaline pH; ambient temperature	[53]
3	Clay/carbon composite	MB	29.54	batch conditions	[51]
4	Activated bentonite clay	MB	22.13	optimized conditions; ambient temperature	[52]
5	Natural Saudi zeolite	MB	24.71	pH 7; 25 °C	[56]
6	Phosphogypsum-tailings-derived zeolite (PGTZ)	MB	31.65	pH 7.42; 25 °C	[57]

## Data Availability

The original contributions presented in this study are included in the article. Further inquiries can be directed to the corresponding author.
